# Association Between DNA Methylation of *MTHFR* and Diabetic Kidney Disease

**DOI:** 10.1155/jdr/8096423

**Published:** 2025-07-15

**Authors:** Guoxiong Deng, Ziyi Feng, Xiaomu Kong, Peng Gao, Yongwei Jiang, Yi Liu, Meimei Zhao, Liang Ma

**Affiliations:** ^1^China–Japan Friendship Institute of Clinical Medicine Research, China–Japan Friendship Hospital, Beijing, China; ^2^Department of Clinical Laboratory, China–Japan Friendship Hospital, Beijing, China

**Keywords:** DKD, DNA methylation, HCY, MTHFR

## Abstract

**Objective:** The objective of this study is to explore the association between *MTHFR* DNA methylation and diabetic kidney disease (DKD).

**Methods:** This study involved 120 healthy people, 200 diabetes mellitus (DM) patients, and 200 DKD patients who visited China–Japan Friendship Hospital from 2022 to 2023. We selected four CpG islands for the detection of *MTHFR* DNA methylation: three located in the promoter region and one in Exon 2. The methylation rate of the *MTHFR* gene was measured using an enzyme digestion method combined with quantitative PCR. Clinical and biochemical characteristics between the two groups were also collected.

**Results:** The methylation rate of the three CpG islands in the promoter region showed no significant differences between the DM and DKD patients. However, a significant difference in the CpG island methylation rate of the *MTHFR* gene Exon 2 was observed (25.14% vs. 21.94%, *p* < 0.001). Logistic regression analysis indicated that the methylation rate of *MTHFR* Exon2 is negatively associated with the occurrence and progression of DKD (OR = 0.947, 95% CI [0.919, 0.977], *p* = 0.001), with adjustments for gender, age, BMI, smoking, drinking, CHO, and TG. Significant differences were observed in the methylation ratios in different HCY groups (24.51% vs. 21.99%, *p* = 0.031). Linear regression showed *MTHFR* Exon 2 methylation negatively correlated with homocysteine (HCY) levels (*p* = 0.007).

**Conclusion:** Methylation of the *MTHFR* gene Exon 2 is a protective factor for DKD and may contribute to its onset and progression through its influence on HCY levels. These findings highlight the potential of *MTHFR* methylation as a biomarker for DKD.

## 1. Introduction

As people's living standards improve, the incidence of diabetes mellitus (DM) is increasing annually. According to the latest 10th edition of the IDF report, the global number of people with DM continues to rise. As of 2021, China has 536.6 million people with DM, with approximately 40% of them having kidney disease [1]. Diabetic kidney disease (DKD) is the most serious complication and the primary cause of death in these individuals.

Despite the high prevalence of DKD among DM patients, diagnosing DKD remains challenging. Currently, the diagnosis mainly relies on renal puncture biopsy and biochemical index detection [2]. Renal biopsy involves using light microscopy, electron microscopy, and immunofluorescence examination to confirm DKD changes, but it is harmful, complicated, and poorly accepted by patients. Therefore, while considered the “gold standard,” renal biopsy is rarely used in clinical practice.

Biochemical tests, such as random urine albumin–creatinine ratio (UACR) or urine albumin excretion rate, are more commonly used to diagnose and stage DKD. However, these indicators are highly influenced by various kidney diseases, necessitating the identification of a more convenient and specific biological marker for DKD [3].

In recent years, epigenetics has emerged as a critical player in the onset and progression of many diseases. Among these, DNA methylation stands out as one of the earliest and most extensively studied epigenetic mechanisms [4]. Its role in DKD has also come to the forefront [5]. Previous research has highlighted significant differences in DNA methylation patterns between DM and DKD, prompting a focus on identifying genes with distinct methylation signatures [6, 7].

Our team has previously identified a link between the C677T gene polymorphism of *MTHFR* and the development of DKD [8]. Furthermore, studies have suggested an association between gene polymorphism and its methylation rate [9], indicating a potential association between *MTHFR* gene methylation and DKD. MTHFR is integral to the folic acid cycle and plays a crucial role in homocysteine (HCY) metabolism, thereby influencing the overall methylation rate in the body. HCY, in turn, is involved in oxidative stress and inflammatory responses [10] and key mechanisms in DKD pathogenesis.

Earlier investigations into kidney tissue methylation in DKD revealed significant differences in *MTHFR* gene methylation rate among cases of simple DM, renal diseases without diabetes, and DKD. Building upon these findings, exploring the association between *MTHFR* gene methylation and DKD holds promise for shedding light on the disease's mechanisms and potentially establishing a specific biological marker for DKD.

## 2. Methods

### 2.1. Participants

We conducted a case–control study on healthy people, DM, and DKD patients who visited the China–Japan Friendship Hospital from 2022 to 2023. Healthy controls were collected from the Physical Examination Department of the China–Japan Friendship Hospital, and all of them had similar baseline data. The DM patients involved in this study were Type 2 diabetes mellitus (T2DM). A total of 200 patients with DM and 200 patients with DKD were included in the screening process. The inclusion and exclusion criteria for DM and DKD were established in accordance with the ADA classification and diagnosis of DM (2023 edition) [11] and the Chinese Guidelines for the Clinical Diagnosis and Treatment of Diabetic Kidney Disease. The details are as follows: inclusion criteria for T2DM: (1) age between 40 and 80 years old, regardless of gender; (2) fasting blood glucose (GLU) ≥ 7.0 mmol/L, random blood GLU or OGTT 2 h blood GLU ≥ 11.1 mmol/L, or HbA1c ≥ 6.5%; and (3) UACR <30 mg/g or estimated glomerular filtration rate (eGFR) >90 mL·min^−1^·(1.73m^2^)^−1^ and inclusion criteria for DKD: (1) age between 40 and 80 years old, regardless of gender, and (2) clear history of T2DM, 3.24-h urine protein > 0.3 g or UACR ≥30 mg/g or eGFR <60 mL·min^−1^·(1.73 m^2^)^−1^ or renal biopsy consistent with DKD pathological changes, twice within half a year.

Patients with the following conditions will be excluded: (1) other serious complications of T2DM; (2) severe macrovascular events in the past 6 months; (3) various infectious diseases in the past 4 weeks, combined with primary and other secondary kidney diseases, combined with other serious diseases, blood system diseases, malignant tumors, etc.; (4) severe psychological or mental abnormalities; and (5) pregnant or lactating women.

### 2.2. Ethical Aspects

This study was approved by the Clinical Research Ethics Committee of the China–Japan Friendship Hospital (Number: 2023-KY-223). The studies were conducted in accordance with the local legislation and institutional requirements. The participants provided their written informed consent to participate in this study.

### 2.3. Data Collection

All participants completed a structured questionnaire to collect information on baseline information, including sex, age, body weight, height, smoking history, and drinking history. Body mass index (BMI) was calculated by dividing weight (kilograms) by height squared (square meter). Enzymatic detection of samples for GLU, HCY, urea, creatinine (Cr), uric acid (UA), cholesterol (CHO), triglycerides (TGs), high-density lipoprotein (HDL), and low-density lipoprotein (LDL) was performed using the corresponding assay kits on the Beckman Coulter AU5800. Immunoturbidimetry was utilized to measure microalbumin (mALB) and the microalbumin/creatinine ratio (mALB/Cr) on the UniCel DxC 800. Hemoglobin A1c (HbA1c) levels were assessed using the Mindray glycosylated hemoglobin meter.

### 2.4. DNA Methylation Detection

#### 2.4.1. Extraction of DNA From Whole Blood Samples

The whole blood sample was first shaken and mixed. Then, 200 *μ*L of whole blood was added to the DNA extraction reagent plate, along with 60 *μ*L of DNA cleavage release reagent and 15 *μ*L of protease. Peripheral blood DNA was extracted using the Tianlong GeneRotex 96 machine. Following extraction, the DNA solution was collected, and its quality was assessed using NanoDrop 2000. It was required that the 260/280 ratio be between 1.8 and 2.0, with a minimum DNA concentration of 20 ng/*μ*L.

#### 2.4.2. Enzymatic Digestion of DNA

**Figure 1:**
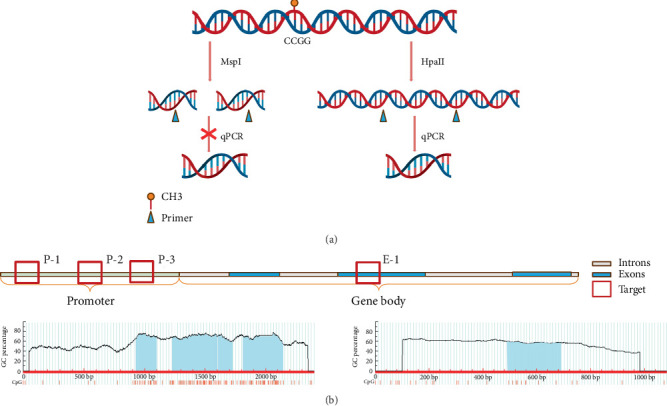
The principle of methylation rate detection and the target region. (a) Principle of enzyme digestion detection. DNA methylation will block the digestion of HpaII, while the digestion of MspI will not be influenced. (b) According to the distribution of CpG islands, we choose four targets to detect. Three of them are in the promoter region (P-1, P-2, and P-3), and one is in Exon 2 (E-1).

The extracted peripheral blood DNA was digested using the Thermo Fisher EpiJET DNA Methylation Analysis Kit (MspI/HpaII). Both MspI and HpaII enzymes recognize the 5⁣′-CCGG-3⁣′ sequence in the gene sequence. When the internal CpG in the 5⁣′-CCGG-3⁣′ tetranucleotide is methylated, cleavage with Epi HpaII is blocked, but cleavage with Epi MspI is not affected. Therefore, the methylation degree of the target fragment can be detected by amplifying and detecting the sequence containing the recognized site (Figure 1a).

To perform enzymatic digestion of DNA for methylation analysis, 200 ng of peripheral blood DNA was taken to mix with 1 *μ*L of MspI/HpaII enzyme and 2 *μ*L of Epi Buffer. Water was added to bring the total volume to 20 *μ*L. A blank control group was also prepared, omitting the enzyme and adding only 2 *μ*L of Epi Buffer. The samples were then incubated in a constant temperature amplification instrument at 37°C for 1 h. After incubation, the samples were heated at 90°C for 10 min to deactivate the enzymes. Finally, the samples were stored at 4°C until further analysis.

#### 2.4.3. Quantitative PCR Analysis

After enzymatic digestion, the DNA was subjected to quantitative PCR analysis. The primers were synthesized by Beijing Qingke Biotechnology Company, and the specific primer sequences are shown in Table 1. Three pairs of primers target the promoter region (P-1, P-2, and P-3), while one pair targets the gene body region (E-1). The reaction system for PCR was prepared using CWbiotech's UltraSYBR Mixture.

The 20 *μ*L system solution was diluted to 200 *μ*L after enzyme digestion. For the PCR reaction, 5 *μ*L of enzyme-digested DNA was mixed with 12.5 *μ*L of the PCR mix and 0.5 *μ*L of upstream and downstream primers to make a 25 *μ*L system.

The experimental procedure was conducted as follows: initial preheating at 95°C for 10 min, followed by 45 cycles of denaturation at 95°C for 15 s, annealing at 50/60°C for 30 s, and extension at 72°C for 30 s. The annealing temperature was set to 56°C for P-1, P-3, and E-1 and 60°C for P-2. This was followed by the melting curve analysis, which included 95°C for 15 s, 60°C for 1 min, and a gradual increase from 60°C to 95°C at a rate of 0.08°C/s, ending with 95°C for 15 s.

#### 2.4.4. Methylation Rate Calculating

When calculating the degree of methylation, the cycle threshold (Ct) value of DNA amplification after MspI digestion is compared with the Ct value of DNA amplification after HpaII digestion. These are then compared to the Ct value of DNA amplification without enzyme digestion, yielding the *Δ*Ct_M_ and *Δ*Ct_H_ values, respectively [12]. The degree of methylation is calculated using the following formula:

 $$\begin{array}{c} \text{Met}=\frac{2^{\wedge }\left(\Delta \text{CtH}\right)}{1-2^{\wedge }\left(\Delta \text{CtM}\right)}*100\% \end{array}$$

### 2.5. Data Analysis

Baseline data were described using standard deviation for normally distributed data and quartiles for nonnormally distributed data. Comparative analysis utilized the chi-square test for categorical variables and the independent sample *t*-test for continuous variables. For data that did not follow a normal distribution, the nonparametric test method (rank sum test) was employed. All data were analyzed using SPSS22.0, with *p* < 0.05 considered statistically significant.

## 3. Results

### 3.1. Baseline Information

Table 2 presents the baseline data for all cases. Significant differences were observed in the levels of HCY, urea, Cr, and UA between patients with DM and those with DKD, suggesting an association between these indicators and the occurrence and progression of renal damage.

### 3.2. Methylation Region Selecting

In this experiment, MethPrimer was utilized for selecting four regions, three in the *MTHFR* promoter region and one in the *MTHFR* gene body, as shown in the diagram below [13]. Four CpG island regions were identified in the *MTHFR* gene promoter region, with lengths of 171 (928–1098), 371 (1230–1600), 116 (1610–1725), and 336 bp (181–2147); GC ratio > 50%; and Obs/Exp > 0.6. Additionally, a CpG island region was found in the promoter region, with a length of 200 bp (486–685), a GC ratio of 55.5%, and Obs/Exp = 0.61 (Figure 1).

### 3.3. Methylation Rate Analysis

Methylation analysis revealed that the methylation rate of the three methylated regions in the gene promoter region of DM patients and DKD patients was 91.14% versus 89.56%, 1.07% versus 1.48%, and 0.59% versus 0.59%, respectively. The *t*-test *p* values were 0.750, 0.124, and 0.570, respectively, all > 0.05, indicating no significant difference in methylation rate in the promoter region. Methylation analysis indicated that the methylation rate of the gene body region (Exon 2) in patients with DM and DKD was 25.14% versus 21.94%, with a *p* value < 0.001 (Table 3). This suggests that the methylation rate of the *MTHFR* gene body may decrease in DKD patients, potentially impacting gene expression.

### 3.4. Association Between Methylation Rate and DKD

In the unadjusted Model 1, logistic regression showed that methylation of MTHFR Exon 2 (Met-E) is negatively associated with DKD (OR: 0.946; 95% CI 0.919–0.947, *p* < 0.001). Considering the influencing factors of DKD and DNA methylation, we included some factors to calibrate the model. In adjusted Model 2, which adjusted for age, sex, BMI, smoking history, drinking history, CHO, and TG, the association between Met-E and DKD still had a significant difference, and the result was similar to Model 1 (OR: 0.947, 95% CI 0.919–0.979). In Model 3, which adjusted for HCY on the basis of Model 2, the association between Met-E and DKD disappeared (OR: 0.964 95% CI 0.918–1.013). Details are shown in Table 4.

### 3.5. Association Between Methylation Rate and HCY

**Figure 2:**
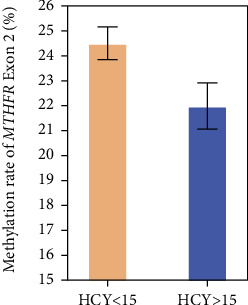
Methylation rate of *MTHFR* Exon 2 in different HCY group patients.

According to the level of HCY, the DM and DKD patients were divided into two groups: one group < 15 *μ*mol/L and one group > 15 *μ*mol/L. The independent sample *t*-test showed a significant difference between the two groups (24.51% vs. 21.99%, *p* = 0.031, Figure 2).

In the unadjusted Model 1, linear regression showed that Met-E was negatively associated with HCY, and in the overall Model 2, which adjusted for age, sex, BMI, smoking history, drinking history, CHO, and TG, this association remained statistically significant (*β* = −0.189, SE = 0.058, and *p* = 0.007). Details are shown in Table 5.

## 4. Discussion

DKD is a severe complication of T2DM, characterized by a complex pathogenic mechanism involving inflammation, fibrosis, and oxidative stress. Physical parameters (such as age and BMI) and environmental factors (such as endocrine disruptors and lifestyle factors including diet, smoking, drinking, and physical exercise) significantly contribute to the development of DKD [14–16], acting via epigenetic modifications such as DNA methylation. DNA methylation typically occurs at CpG islands and can alter the chromatin structure of genes, thereby influencing their expression levels [17]. Depending on its location, methylation can have varying effects on gene regulation. Methylation within gene promoters generally suppresses gene expression, whereas methylation within gene bodies often enhances it [18]. Although the mechanism and influence of gene methylation on gene expression and function are complicated and require comprehensive investigation [19], the methylation biomarkers show a great potential for disease prediction and monitoring in clinical practice [20].

In the present study, baseline data analysis revealed significantly higher serum HCY levels in DKD patients compared to DM patients (17.31 vs. 11.36, *p* < 0.001). Although differences were also observed in the baseline data for urea and Cr, as these indicators are directly related to kidney function, no further analysis was performed. However, significant differences were noted in UA baseline data, aligning with findings from earlier studies. UA has been shown to correlate with DKD and holds potential as a predictive marker for the disease [21]. Nevertheless, no significant association was found between the methylation rate of the *MTHFR* gene and UA levels.

As the key sites where methylation occurs, CpG islands play the essential role in the epigenetic regulation of gene expression. In order to investigate the association between *MTHFR* gene methylation and DKD, we conducted a comprehensive analysis to identify CpG islands in *MTHFR* gene promoter and gene body. The analysis finally identified three CpG islands within the promoter region and one within Exon 2, all of which contain 5⁣′-CCGG-3⁣′ sequence that can be recognized by MspI/HpaII used in this method. The findings revealed no significant differences in the methylation rate at CpG sites within the promoter region of the *MTHFR* gene. However, within the gene body, specifically in Exon 2, DKD patients exhibited reduced methylation compared to DM patients (21.94% vs. 25.14%, *p* < 0.001). Besides, the DNA methylation level of controls has significant difference between DM and DKD, which means that it has the potential to be used for biomarker. We pay attention to the association between DM and DKD and conducted further analysis. Then, logistic regression analysis demonstrated that the reduced methylation rate of *MTHFR* Exon 2 is associated with the increased risk of DKD (OR: 0.964, 95% CI 0.918–1.013, *p* = 0.001). These indicated that *MTHFR* Exon 2 methylation may serves as a protective factor against DKD. After adjusted for HCY, the association between Met-E and DKD diminished (*p* = 0.146). It is suggested that DNA methylation may influence the DKD via HCY. Further, we found significant differences in the methylation rate of *MTHFR* Exon 2 between patients with different HCY levels (24.51% vs. 21.99%), further proving that there is a correlation between HCY and the methylation rate of *MTHFR* Exon 2. In addition, there were several previous studies attempted to explore *MTHFR* gene methylation in DKD. A study found higher *MTHFR* promoter region methylation in DKD patients compared to DM patients, inhibiting *MTHFR* expression, elevating HCY levels, and promoting DKD development [22]. While another study has conducted similar research, indicating that as the disease progresses from simple DM to prediabetic nephropathy, and ultimately to clinical diabetic nephropathy, the methylation rate of the *MTHFR* promoter region gradually decreases, eventually becoming completely unmethylated [23]. First, these discrepant results can be reasonably explained by the different CpG sites examined. The targeted CG fragments in previous studies differ from the sites examined in this study. In fact, methylation status of CpG islands and their downstream biological effects is very complicated. Even within a certain gene region, the methylation levels of different CpG sites can be different, and their changes in disease status vary. Thus, a specific CpG site is unable to represent the entire gene's methylation status [24]. Moreover, unlike the previous studies that focused exclusively on the gene's promoter region, we also involved CpG island in Exon 2 in addition to promoter region. Second, the discrepant results may arise due to different methylation detecting methodologies applied. Methylation-specific PCR (MSP) that performed in the previous two studies is bisulfite conversion based. The digestion-based methylation detection method used in the present study does not rely on bisulfite conversion sequences, making it less destructive to gene sequences and resulting in more stable outcomes [25]. By employing an enzyme digestion method [26] combined with quantitative PCR, the study achieved enhanced specificity and accuracy compared to previous gel-based methods [27]. This approach provides a more reliable assessment of the methylation status of the *MTHFR* gene in DKD patients. Finally, the limited sample size and source of the study population, as well as the different features of the population (such as race, age, lifestyle, and other environmental exposures), could contribute to the discrepancies.

According to our research, HCY plays an important role in the occurrence and development of DKD. As an inflammatory factor, HCY has two main metabolic pathways in the body. First, HCY is catalyzed by MTHFR and converted into methionine, which is further converted into S-adenosylmethionine (SAM), a key methyl donor in methylation reactions [28]. Second, HCY is degraded by cystathionine beta-synthase (CBS) into cystathionine, which is subsequently converted into cysteine and eventually into glutathione, playing a role in oxidative stress pathways [29]. Therefore, HCY can participate in inflammatory responses, methylation changes, and oxidative stress pathways. These pathways all contribute to the occurrence and development of DKD. Previous studies have identified HCY as an independent risk factor for DKD [30]. What is more, a predictive study using HCY levels as a predictor for DKD development has shown promising results [31]. In addition, elevated folic acid level has been found to ameliorate the adverse effects of high HCY, which is closely linked to *MTHFR* gene function in patients with DKD [32]. HCY may serve as a bridge between *MTHFR* and DKD, influencing the pathological process of DKD by modulating HCY levels.

The reduction of DNA methylation in the *MTHFR* gene Exon 2 may decrease *MTHFR* expression and content. Since MTHFR is crucial for HCY metabolism, reduced expression could lead to elevated HCY levels, thereby increasing the inflammatory response, potentially contributing to DKD development. Association analysis further indicated a negative association between the *MTHFR* Exon 2 DNA methylation rate and HCY levels.

In addition, it is well-established that multiple environmental risk factors (including smoking, drinking, exercise, and age increasing) contribute to DKD [5]. Researchers also reported gene methylation could be mediated by environmental factors (such as diet and smoking drinking) [33]. Several studies indicated that physical parameters and environmental factors significantly contribute to the development of DKD, acting via epigenetic modifications such as DNA methylation [34]. Notably, one study indicated that the methylation status of the *MTHFR* gene can be influenced by diet and smoking in pregnant women [35]. Based on these findings, we speculated that the altered methylation status of the *MTHFR* gene attributed to environmental factors could also play a significant role in DKD. However, whether the environmental factors could influence the risk for DKD via mediating the MTHFR methylation status is still unknown and warrants investigation.

The association among DKD, *MTHFR*, and HCY has done some research before. Previous studies indicated that hypomethylation of the *MTHFR* promoter region may result in increased MTHFR expression [23]. The authors suggest that the elevated MTHFR expression is a consequence of negative feedback triggered by the abnormal rise in HCY. From another perspective, our study posits that the decrease in *MTHFR* gene methylation leads to increased MTHFR expression, subsequently elevating HCY levels and ultimately contributing to the development of DKD.

This study also has some limitations. Firstly, as a single center-based study in a population of Chinese ancestry, the results were limited for generalization. Studies with larger sample sizes are required for further validation. Secondly, as a case–control study, it revealed an *MTHFR* methylation site significantly associated with DKD, but cannot identify temporal or causal relationships. A longitudinal study is required to explore the causative effect of *MTHFR* methylation on DKD pathogenesis. Finally, the present method detected specific sites which may cause some omissions, but it is a stable, convenient, and precise technique for methylation detection. Overall, the findings provide valuable data for exploring novel methylation biomarkers for DKD.

## 5. Conclusion

Differences in *MTHFR* Exon 2 methylation rate between DM patients and DKD patients were observed. The methylation rate of the *MTHFR* Exon 2 was negatively correlated with DKD occurrence and HCY levels. This research suggests that reduced *MTHFR* Exon 2 methylation rate may influence DKD development by elevating HCY levels. However, further basic experiments are required to confirm the association between these factors.

## Figures and Tables

**Table 1:** Primer sequence information.

	**Forward primer**	**Reverse primer**	**Product**
P-1	5⁣′-AGCAGGGTAGACGCTTCGAG-3⁣′	5⁣′-AGGGGTCCAGAGTGGCAGTA-3⁣′	272 bp
P-2	5⁣′-CGCGTCACATGACGATAAAGG-3⁣′	5⁣′-GCCAAAGTCAGTCTTCGCTTG-3⁣′	450 bp
P-3	5⁣′-ACCTGGTGACTGGATTCTCG-3⁣′	5⁣′-CCTGGGTTGTAACTGTGGGT-3⁣′	498 bp
E-1	5⁣′-GGTGAACGAAGCCAGAGGAA-3⁣′	5⁣′-CCACCTTAACCTTGCATGAGT-3⁣′	259 bp

**Table 2:** Baseline level information.

**Variables**	**Control**	**DM**	**DKD**	**p**
Gender (M:F)	47:73	112: 88	148: 52	< 0.001
Age (years)	54.07 ± 10.10	61.39 ± 7.93	62.16 ± 10.02	0.398
BMI (kg/m^2^)	NA	25.47 ± 4.10	25.94 ± 3.54	0.220
Smoking history	NA	64 (32.0%)	80 (40.0%)	0.076
Drinking history	NA	52 (26.0%)	70 (35.0%)	0.039
HCY (*μ*mol/L)	9.89 ± 2.33	11.36 ± 3.24	17.31 ± 7.36	< 0.001
Urea (mmol/L)	4.80 ± 1.37	5.69 ± 1.53	11.89 ± 7.96	< 0.001
Cr (*μ*mol/L)	67.54 ± 15.09	63.05 ± 14.06	128.80 (86.50, 216.73)	< 0.001
eGFR (ml/min/1.73m^2^)	100.78 ± 15.08	96.23 ± 11.21	50.85 ± 31.84	< 0.001
UA (*μ*mol/L)	298 ± 77.72	348.31 ± 85.71	384.53 ± 103.15	< 0.001
GLU (mmol/L)	5.28 ± 0.39	8.23 ± 2.81	8.71 ± 3.52	0.136
CHO (mmol/L)	4.99 ± 1.11	4.04 ± 1.13	4.17 ± 1.32	0.294
TG (mmol/L)	1.34 ± 0.93	1.65 ± 0.92	1.82 ± 0.99	0.073
HDL-C (mmol/L)	1.137 ± 0.37	1.21 ± 0.31	1.19 ± 0.34	0.649
LDL-C (mmol/L)	2.91 ± 0.94	2.45 ± 0.81	2.53 ± 0.91	0.340
HbAlc (%)	NA	7.44 ± 1.55	7.61 ± 1.74	0.320
mALB (mg/L)	NA	0.10 (0.10, 6.95)	343.17 (86.16, 687.7)	< 0.001
mALB/Cr (mg/g)	NA	0.16 (0.10, 5.48)	382.84 (97.28, 1126.48)	< 0.001

Abbreviation: NA, not available.

**Table 3:** Methylation rate of control, DM, and DKD patients.

**Target**	**Control**	**DM**	**DKD**	**p** ** (DM vs. DKD)**
P-1	50.27% ± 2.18%	91.14% ± 3.54%⁣^∗^	89.56% ± 3.45%⁣^∗^	0.750
P-2	0.56% ± 0.04%	1.07% ± 0.16%⁣^∗^	1.48% ± 0.21%⁣^∗^	0.124
P-3	0.28% ± 0.02%	0.59% ± 0.10%	0.59% ± 0.11%	0.570
E-1	32.85% ± 0.70%	25.14% ± 0.57%⁣^∗^	21.94% ± 0.61%⁣^∗#^	< 0.001

⁣^∗^*p* < 0.01 versus control; ^#^*p* < 0.01 versus DM.

**Table 4:** Logistics regression analysis of Met-E and DKD.

	**OR**	**Lower 95%**	**Upper 95%**	**p**
Model 1	0.946	0.919	0.947	< 0.001
Model 2	0.947	0.919	0.977	0.001
Model 3	0.964	0.918	1.013	0.146

*Note:* Model 1: not adjusted; Model 2: adjusted for age, sex, BMI, smoking history, drinking history, CHO, and TG; Model 3: adjusted for age, sex, BMI, smoking history, drinking history, CHO, TG, and HCY.

**Table 5:** Linear regression analysis of Met-E and HCY.

	**β**	**SE**	**p**
Model 1	−0.195	0.057	0.005
Model 2	−0.189	0.058	0.007

*Note:* Model 1: not adjusted; Model 2: adjusted for age, sex, BMI, smoking history, drinking history, CHO, and TG.

## Data Availability

All data generated or analyzed throughout this study are included in this article.
